# P-1220. Analyzing trends of cefiderocol susceptibility across five years of the SENTRY surveillance program

**DOI:** 10.1093/ofid/ofaf695.1413

**Published:** 2026-01-11

**Authors:** Yoshinori Yamano, Boudewijn L DeJonge, Sean T Nguyen, Jason Bryowski, Joshua Maher, Rodrigo E Mendes, Hidenori Yamashiro, Christopher M Longshaw

**Affiliations:** Shionogi & Co., Ltd., Toyonaka, Osaka, Japan; Shionogi Inc., Florham Park, NJ; Shionogi Inc., Florham Park, NJ; Shionogi Inc., Florham Park, NJ; Element Materials Technology/Jones Microbiology Institute, North Liberty, Iowa; Element Iowa City (JMI Laboratories), North Liberty, IA; Shionogi & Co., Ltd., Toyonaka, Osaka, Japan; Shionogi B.V., London, England, United Kingdom

## Abstract

**Background:**

Cefiderocol (FDC) is a siderophore-conjugated cephalosporin having activity against Gram-negative pathogens including carbapenem-resistant strains. It was approved for clinical use in the USA in 2019 and in Europe in 2020. We have assessed the *in vitro* activity for FDC against isolates that were collected between 2020-2024 in Europe and the USA, after it was introduced onto the market, as part of the SENTRY Antimicrobial Surveillance Program. In this study, trends in FDC susceptibility across five years were analyzed.

**Figure 1.**
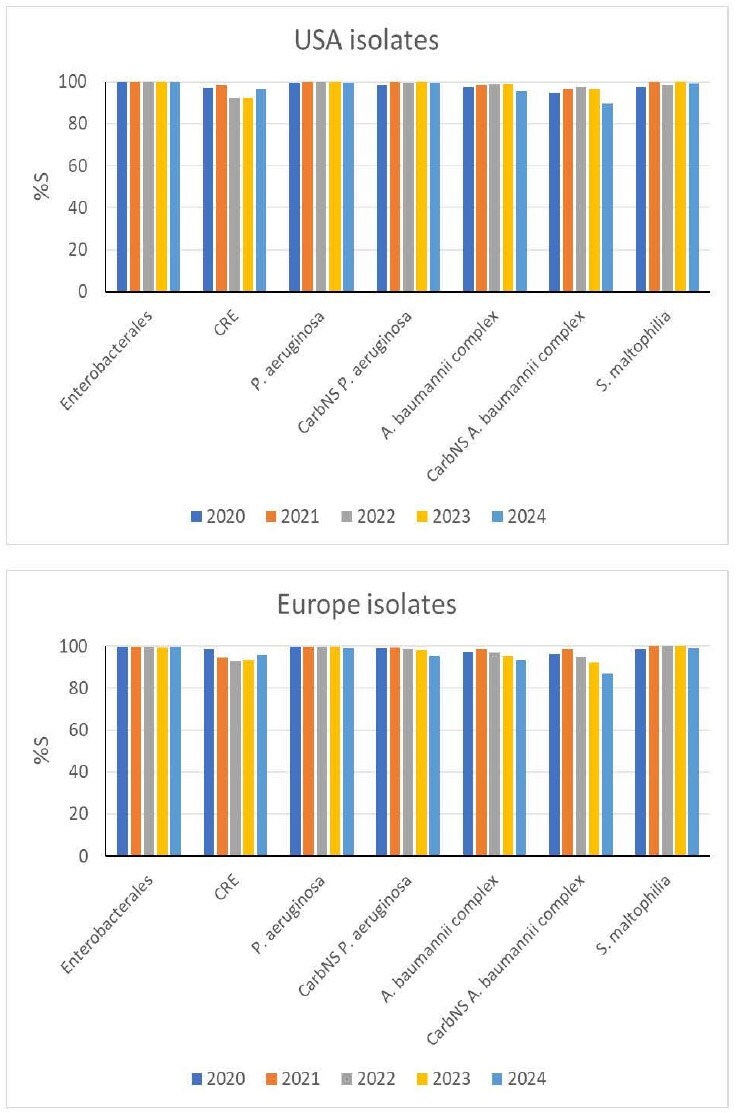
Activity for cefiderocol by year against Gram-negative isolates collected from US and European hospitals as part of the SENTRY surveillance program (2020-2024). The susceptibility percentage (%S) by year was determined based on the CLSI breakpoints for each bacterial species including carbapenem-resistant or -non-susceptible subsets.

**Methods:**

A total of 60,582 isolates were collected and minimum inhibitory concentrations (MICs) for FDC were determined according to CLSI guidelines using broth microdilution with iron-depleted CAMHB. Susceptibility to cefiderocol and carbapenems were defined using 2025 CLSI breakpoints.

**Results:**

Among a total of 60,582 collected isolates, 40,048 were Enterobacterales, 11,757 were *Pseudomonas aeruginosa*, 4,806 were *Acinetobacter baumannii* complex, and 2,346 were *Stenotrophomonas maltophilia*. Susceptibility trends in each region (the USA and Europe) across five years were assessed for these species (Figure). Overall, consistent high activity was demonstrated for FDC against these bacterial species across five years. The susceptibility percentage (%S) based on the CLSI breakpoints was ≥99.5% for Enterobacterales and ≥92.3% for carbapenem-resistant isolates irrespective of region. The %S based on the CLSI breakpoint was 98.8-100% for all *P. aeruginosa* and 95.3-100% for the carbapenem-nonsusceptible (CarbNS) subsets. The lowest %S (95.3%) was observed for CarbNS *P. aeruginosa* in Europe in 2024. The %S based on CLSI breakpoint was 93.2-98.9% for all *A. baumannii* complex and 87.0-97.7% for CarbNS subset. The %S lower than 90% (89.4 and 87.0%) were observed for CarbNS *A. baumannii* complex in the USA and Europe in 2024, respectively. The %S based on the CLSI breakpoint was 97.2-100% for all *S. maltophilia*.

**Conclusion:**

Consistent high activity for FDC against Gram-negative isolates, including carbapenem non-susceptible subsets, was observed across five years, after FDC was introduced onto the market. Continued monitoring of FDC susceptibility is warranted to assure its activity is maintained over time.

**Disclosures:**

Yoshinori Yamano, PhD, Shionogi HQ: Employee Boudewijn L. DeJonge, PhD, Shionogi Inc.: Employee Sean T. Nguyen, PharmD, Shionogi Inc: Employee Rodrigo E. Mendes, PhD, GSK: Grant/Research Support|Shionogi & Co., Ltd.: Grant/Research Support|United States Food and Drug Administration: FDA Contract Number: 75F40123C00140 Hidenori Yamashiro, Shionogi HQ: Employee Christopher M. Longshaw, PhD, Shionogi BV: Employee

